# Are ophthalmologists being trained for Brazil’s social needs?

**DOI:** 10.6061/clinics/2020/e2201

**Published:** 2020-11-02

**Authors:** Newton Kara-Junior

**Affiliations:** Disciplina de Oftalmologia, Faculdade de Medicina (FMUSP), Universidade de Sao Paulo, SP, BR

Considering the characteristics and social needs of Brazil, I believe that most of our ophthalmologists should meet the following requirements:

- Be homogeneously distributed throughout the national territory to facilitate access for most of the population in their care; and
- Be a generalist able to treat approximately 90% of eye problems, using few complementary exams at a cost that the community can bear.

Subspecialist ophthalmologists are important, but in limited capacity, to receive referrals for complex cases that a well-trained generalist specialist may not be able to solve (1). Subspecialists, in general, depend on advanced technology and work in large urban centers. Their expertise, when called on more frequently than necessary, leads to more expensive treatment and raises difficulties in the access to treatment (2).

The Ministry of Education (MEC) and the Brazilian Council of Ophthalmology (CBO), which is part of the Brazilian Medical Association, have the function of regulating specialty training and conferring the RQE (Registry of Specialist Qualification), which is the official recognition of specialization, a necessary condition for the doctor to practice as an ophthalmologist. However, in Brazil, every trained doctor can practice any medical specialty, regardless of their specialization and having the RQE. Thus, it is possible that many doctors, excluded from official specialization opportunities, practice ophthalmology right after graduation or look to practice in institutions not accredited by the MEC or CBO. These institutions are often ill-prepared to offer training of adequate quality. Although these professionals cannot be called ophthalmologists, they can work in eye healthcare because they have a medical degree.

This scenario, which we believe has been occurring more and more frequently, due to the large number of recently graduated doctors, leads to doctors working in the field of visual health without appropriate training. We imagine that this professional can then follow one of several paths:

- To act as an independent professional outside or on the periphery of a large urban center in a region where a doctor’s clinical capability tends to be little contested;
- To work as a collaborator in the screening of cases in private health institutions, in general, screening and referring cases for subspecialists to treat, and
- To invest more time in training and supplement that training by pursuing a subspecialty fellowship.

We believe that some doctors “specialized” in unofficial institutions, which are usually unprepared for teaching, try to subspecialize to compensate for basic clinical deficiencies in training and to obtain expertise in at least one segment (3). In the subspecialization model, knowledge is divided into specific areas, which produces a lack of communication between specialties (4-7). Thus, while the specialization program ideally includes general discussions and focuses mostly on the entire specialty, which allow a panoramic view of ophthalmology, this type of discussion, in general, does not happen at the stage of subspecialization, where teaching is directed to a single segment.

Subspecialists, in the above conditions, even manage to be good surgeons or performers/interpreters of supplementary exams, but they do not have adequate general training that allows them to work outside the group of a segmented service system. Thus, although they extend their training time, they are not adequately trained and become hostage to market circumstances when trying to obtain work.

In the social context, the excess of subspecialists makes medicine more expensive, because the characteristics of their training, leads subspecialists to recommend many supplementary exams for their patients and to prescribe excessively complex treatments. In addition, by working in large urban centers, they contribute to worsening the already uneven geographical distribution of ophthalmologists across the country.

Thus, we believe that one of the consequences of inadequate specialized training is the subspecialization of some ophthalmologists, not because of an interest in the subject or job prospects, but because of a lack of technical learning.

Another problem resulting from inadequate specialized training is that, if the training of the generalist “ophthalmologist” is not adequate, the doctor can tend to feel a lack of clinical efficiency (resolvability) and has to excessively refer patients to a subspecialist, making the treatment more difficult and more expensive.

At the current rate of subspecialist training, which we observe to be disproportionate to social needs, in the near future, many subspecialist ophthalmologists, mainly in large urban centers, may have to choose to work for a health plan or for a large institution (8-10). To act as a liberal professional in a private practice, the subspecialist must have good quality specialization training and must also be a good generalist.

We believe that ophthalmologists with good general clinical training, trained for a surgical modality, have a good chance of succeeding as a professional in any part of the country, as they will be prepared to solve most of their patients’ visual problems with clinical reasoning and with little technology. Although subspecialists train predominantly in a specific area of expertise, they are unlikely to experience good working conditions away from large urban centers with advanced (and expensive) technology.

As a result, we consider that the deficiency in the specialization training of doctors who wish to work as ophthalmologists ultimately makes treatment more expensive and makes access to eye healthcare more difficult.

## AUTHOR CONTRIBUTIONS

Junior NK contributed in studied, reflected and writing of the text.
